# Reduced-order modeling of soft robots

**DOI:** 10.1371/journal.pone.0192052

**Published:** 2018-02-22

**Authors:** Jean Chenevier, David González, J. Vicente Aguado, Francisco Chinesta, Elías Cueto

**Affiliations:** 1 Aragon Institute of Engineering Research, Universidad de Zaragoza, Zaragoza, Spain; 2 ESI Group chair at Ecole Centrale Nantes and the High Performance Computing Institute, Nantes, France; 3 PIMM, ENSAM ParisTech. Paris, France; University of Manchester, UNITED KINGDOM

## Abstract

We present a general strategy for the modeling and simulation-based control of soft robots. Although the presented methodology is completely general, we restrict ourselves to the analysis of a model robot made of hyperelastic materials and actuated by cables or tendons. To comply with the stringent real-time constraints imposed by control algorithms, a reduced-order modeling strategy is proposed that allows to minimize the amount of *online* CPU cost. Instead, an *offline* training procedure is proposed that allows to determine a sort of *response surface* that characterizes the response of the robot. Contrarily to existing strategies, the proposed methodology allows for a fully non-linear modeling of the soft material in a hyperelastic setting as well as a fully non-linear kinematic description of the movement without any restriction nor simplifying assumption. Examples of different configurations of the robot were analyzed that show the appeal of the method.

## Introduction

Originally, soft robots are born from a biological inspiration, to reproduce some living being’s compliance, see [1] [2] [3]. There exists a wide spectrum of application fields of soft robotics as well as the crowd of engineering issues they raise, in terms of design, fabrication and control. Examples exist of pneumatically actuated soft robots, such as [4], and thus without any type of “skeleton”, as well as hydraulic ones [5]. Maybe the biggest family of soft robots is the one actuated by cables or tendons, [1]. But the main concern with the design, modeling and control of this type of robots is clearly motivated by the passage from a discrete to infinite number of degrees of freedom. In other words, the difference with classical, rigid robots is the same that exists between rigid solids and deformable, continuum solids. Therefore, one major difficulty arises when one tries to model the relationship between the actuators and the effectors, since this response is often highly non-linear, on one side, and is required under severe feedback restrictions (real time), on the other.

Contact with other objects during motion also poses major difficulties to the problem at hand. This is so since contact is a highly non-linear problem, governed by Kuhn-Tucker conditions [6]. It was not until very recently that an inverse method of control based on simulation and considering contact has been presented in [7]. In this paper we propose a methodology that abandons completely existing strategies based on real time (usually, finite element-based) simulation nor employs any kinematical assumption that eventually allows to simplify the problem.

Indeed, the presented methodology is based upon the *offline* construction of a response function for the robot. This response function or computational *vademecum* is stored in memory as a finite sum of products vectors with a minimum amount of memory and re-constructed online with negligible computational cost [8]. Therefore, our proposal is based in *evaluating* the response of the robot rather than *simulating* the response of the robot.

In fact, our proposed methodology is aimed at describing the control problem as an inverse problem arising from a parameterized partial differential equation (PDE), and thus is amenable to generalization to virtually any type of soft robot. As will be noticed throughout the paper, our strategy allows for an efficient inverse determination of the necessary parameters (here, the forces in the actuators) given stress or pressure limitations at the effectors after contact. Simple Levenberg-Marquardt algorithms provide results compliant with the desired interactive rates (from some 30 to 130 Hz in our experiments with code prototypes).

## Related work

As mentioned before, the passage from discrete to continuum makes modeling and simulation of soft robots an intricate procedure that strongly depends on the considered type of robot. In order to overcome the infinite-dimensional configuration space of continuum mechanics, recent approaches try to follow the tradition of the fathers of strength of materials disciplines, i.e., to establish some kinematic assumptions that help to alleviate the complexity of the problem. Thus, for instance, in [9], a procedure is established in which piecewise constant curvature is assumed for each of the segments of a pneumatic actuator. Similarly, in [10], a pressure-volume relationship is constructed for a soft, hydraulically-actuated robot able to transverse a cannula. This is also the approach followed in [11], where the Cosserat rod theory was applied to modeling a soft robot arm driven by cables, similar to the one considered here. However, in sharp contrast with the approach followed herein, in that work a linear visco-elastic (Kirchhoff-Saint Venant) model is considered, which can lead to severe inconsistencies (particularly, crushing under compression) [12].

A second group of techniques employs finite element modeling under real-time constraints. To fulfill these constraints, usually some simplifying assumptions are made. In [13], for instance, FEM is employed to characterize an octopus-like soft robot guided by cables and springs. This concept is further generalized in [14] to coin the concept of *eRobotics*, i.e., a virtual testbed for the design, modeling and simulation-based control of soft robots.

The work of C. Duriez and coworkers is maybe the most relevant concerning real-time finite element simulation for control of soft robots, see [15] [7] [16]. In his work, although non-linear, explicit finite element methods are used, some severe simplifications are taken into account. For instance, given the impossibility of performing inverse analysis in the displacement space, due to the high number of degrees of freedom, they opt by doing it in the actuation and contact variables. Linear elasticity under the corotational FE framework is employed at a first step. In sharp contrast with these assumptions, in our method general hyperelastic laws can be employed at no extra cost.

As a result of this first step, the model in [15] could eventually violate the contact restrictions. In parallel, a second problem is solved in which a linear relationship between the actuations and the contact forces is solved. The deformed configuration of the robot would thus be the sum (linear superposition) of the unconstrained motion of the robot and the constraints motivated by contact. Forces in the actuators are thus obtained by juxtaposition of both problems, despite the (theoretical) non-linearity of both and the lack of fulfillment of the superposition principle.

Despite these limitations, the work by Duriez and coworkers, see the original one in [15] and a very recent update in [7], is perhaps the most sophisticated method based on the finite element method. This proves the inherent difficulty of the problem at hand.

## Method

The method we present here is aimed at overcoming the mentioned simplifications motivated by the complexity (and the high number of degrees of freedom) of standard finite element approaches to modeling and control of soft robots. The goal is to consider a model of the robot as general as possible, and to that end we chose a tendon-driven finger, which under similar forms appears in different references, see [11] [13] [15], among others. No particular assumption is made on the linearity of the constitutive equation of the matrix, and the control strategy is also extensible to any robot whose control can be set as an optimization problem arising from a parametrized partial differential equation (PDE).

### Abstract setting

We formulate the problem of control of the soft robot as the fast evaluation of the response of the system, whose output of interest is expressed as some linear functional of a field variable (typically, the displacement field), that is the solution of a parametrized partial differential equation (PDE). This evaluation must be also bounded in terms of error for the strategy being of practical interest, of course.

**Fig 1:**
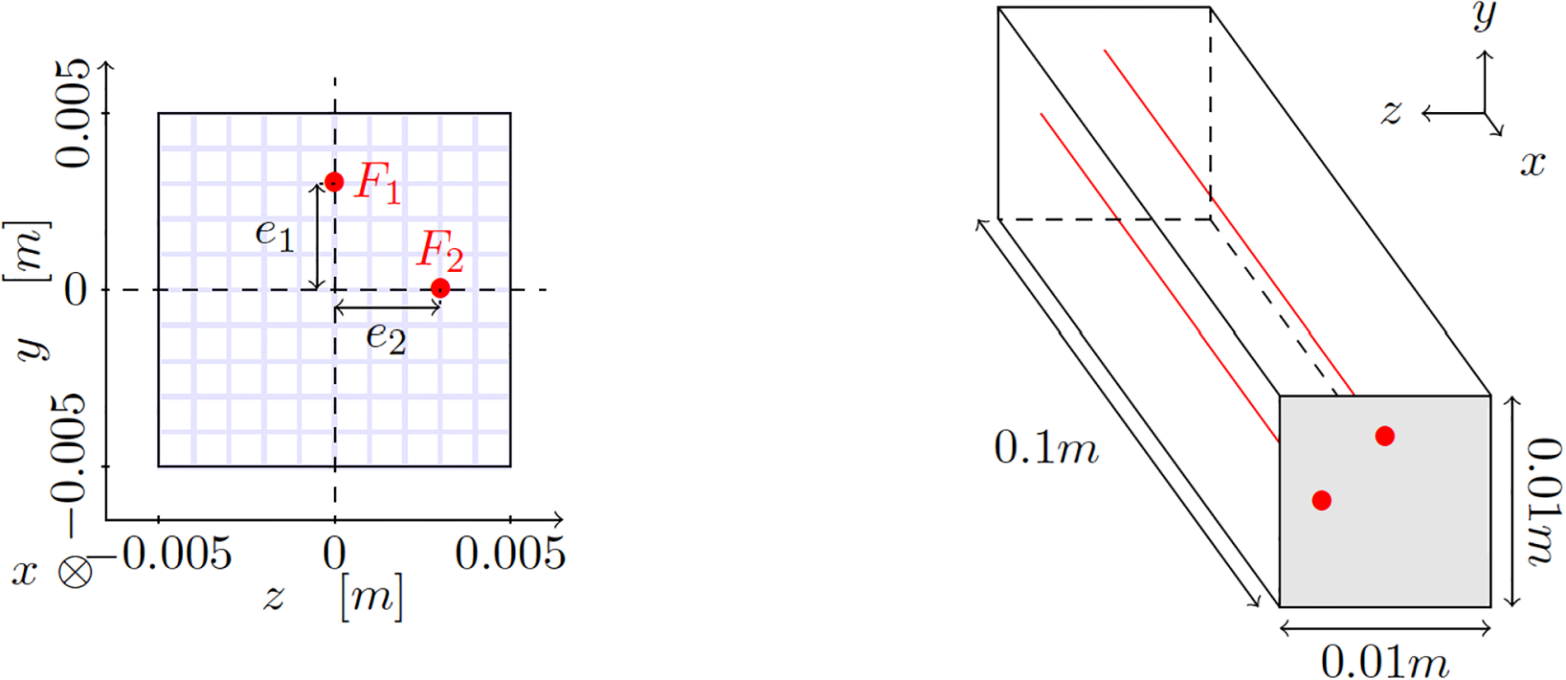
Schematic description of the tendon-driven manipulator considered in this work, along with its dimensions. Left, cross section of the segment with the position of the tendons. Right, dimensions of the segment. Due to symmetry, and to avoid degenerate solutions, we consider that only two tendons can be actuated at a time. These are represented in red. Other possible configurations can be obtained by just rotating around the *x*-axis the solution obtained for this particular one. Actuating the four tendons at the same time could produce, for instance, a pure compressive stress state that would produce a shortening of the finger, something useless, in general.

To better describe our approach, consider without loss of generality, a model robot inspired by the Clemson manipulator (essentially, a tendon driven continuum manipulator) [17]. A similar robot has been considered recently in [7], for instance. The robot can be composed by one or more segments, each of them actuated by four tendons (steel cables), see Fig 1. The tendons are attached to a rigid plate (represented in grey in Fig 1) placed at the end of the actuator, so as to transmit their tension and provoke bending.

The response of the robot, still without considering contact, will then typically take the form of a function

u=u(x,μ), (1)

where ***u*** represents the vector-valued field of displacements at any point ***x*** of the volume Ω_*t*_ = Ω(*t*) occupied by the robot. Here, $\mu \in R^{n_{\text{par}}}$ represents a vector of $n_{\text{par}}$ parameters governing the behavior of the robot. For the tendon-driven manipulator in Fig 1, these parameters will be the forces in both tendons, i.e., ***μ*** = [*F*_1_, *F*_2_]. For other types of soft robots such as pneumatic ones, these parameters could be pressures at different points of the robot Ω, for instance.

The just mentioned displacement field given in Eq (1) will be the solution of the equilibrium equations for the robot, i.e.,

$$\nabla P+B=0\;\;\;\text{in}\;\Omega_{0}$$

where ***B*** represents the volumetric forces applied to the body and ***P*** the first Piola-Kirchhoff stress tensor. Ω_0_ = Ω(*t* = 0) represents the undeformed configuration of the robot. The solution is subjected to the following boundary conditions

$$\begin{array}{c} u\left(X\right)=\bar{u}\;\;\text{on}\;\Gamma_{u},\\ PN=\bar{t}\;\;\text{on}\;\Gamma_{t} \end{array}$$

Γ_*u*_ and Γ_*t*_ represent the essential (Dirichlet) and natural (Neumann) portions of the boundary Γ = ∂Ω of the robot. ***N*** is the unit vector normal to Γ = ∂Ω_0_ and $\bar{t}$ is an applied traction. To complete the problem, some relationship between kinematic variables (displacements, strain) and dynamic variables (stresses) must be assumed. Here, it is assumed that the material is composed by a neo-Hookean, and thus hyperelastic, material, see [12], although any other hyperelastic constitutive law could be considered without any difficulty. Its strain energy density function is defined as

$$W=C_{1}\left(I_{1}-2\right)+D_{1}{\left(J-1\right)}^{2}.$$

Here, we take *C*_1_ and *D*_1_ are constants, characteristic of the particular material employed. *I*_1_ represents the first invariant of the isochoric part of the right Cauchy-Green deformation tensor and *J* is the determinant of the gradient of deformation tensor.

Therefore, as can readily be noticed, the problem greatly simplifies if we are able to compute *offline* the response of the system, Eq (1), and to evaluate it *online*, rather than simulate it by standard finite elements, for instance. Under this rationale, we call *direct* problem the straightforward obtention of the displacement field —or any related quantity of interest (QoI) given by a linear functional *ℓ*^*o*^(***u***) such as the displacement at the end effector, for instance—, given the values of the parameters, ***μ***. Generally, however, control strategies will involve *inverse* problems, i.e., given the desired QoI, find the right values of the parameters ***μ*** that provide it.

Solving in real time the inverse problem will pose, unless artificial linearity assumptions are made, very stringent requirements to the control strategy. In general, despite the nowadays capabilities of modern computers or even deployed systems that could be installed, this is still out of reach for realistic models of soft robots.

### Reduced order modeling of the soft robot

Although many different model order reduction (MOR) techniques exist (see, for instance, some recent reviews in [18], [19], [20], [21], [22], to name but a few), their main characteristic is to provide a model with a minimal number of degrees of freedom with also minimal loss in accuracy. This accuracy is often compared to what is called *full-order models*, i.e., detailed finite element models of the system of interest, in our case.

To achieve this, MOR techniques employ different methods for generating an *affine* or *separated* representation of the unknowns, viz. the displacement filed in our model problem:

$$u\left(x,\mu \right)\approx \sum_{i=1}^{n_{\text{DOF}}}F_{i}\left(x\right)\circ G_{i}^{1}\left(\mu_{1}\right)\circ G_{i}^{2}\left(\mu_{2}\right)\circ \ldots \circ G_{i}^{n_{\text{par}}}\left(\mu_{n_{\text{par}}}\right),$$ (2)

where “∘” appears here as the Hadamard entry-wise product of vectors (Matlab “*.” product), given the vectorial character of the displacement field. Functions ***F***_*i*_ and ***G***_*i*_ are actually expressed in a finite element mesh and must be determined so as to provide the model with a minimal number of degrees of freedom $n_{\text{DOF}}$. Many MOR methods employ a *learning* phase in which snapshots of the full-order model for different parameter values are computed. Then, an *a posteriori* analysis of these snapshots provides the optimal functions ***F***_*i*_ and ***G***_*i*_. Since they are expressed in a finite element mesh of low dimension, their storage in memory and subsequent reconstruction of the solution (2) for a given value of the parameters ***μ*** is straightforward. Among these methods one can cite the plethora of techniques based on Proper Orthogonal Decomposition (POD) [23] [24] [18] or the Reduced Basis technique [20] [25].

Proper Generalized Decompositions (PGD), however, compute these functions *a priori*, and thus without the need of any learning campaign [26] [27]. To that end, PGD methods employ a greedy algorithm to compute each term in the sum (2). Within each loop *i* in this greedy algorithm, the usual procedure to obtain the (nodal values of) the functions ***F***_*i*_ and ***G***_*i*_, given the non-linear character of the product, is to employ Newton iterations or, more commonly, simple fixed-point alternating directions algorithms.

Standard finite element approximations to the displacement field ***u***(***x***, ***μ***) are usually out of reach, due to the high dimensional space in which it lives. Indeed, the phase space of the problem, given that $x\in R^{3}$ and that, in general, $\mu \in R^{n_{\text{param}}}$, will be defined in $R^{3n_{\text{param}}}$. The number of degrees of freedom of a finite element mesh in a high-dimensional space is known to grow exponentially with the number of dimensions, and therefore will render the method useless for a moderate number of parameters $n_{\text{param}}$. However, reduced-order models keep the number of degrees of freedom moderate. From Eq (2) we observe that the total complexity of the problem scales linearly (and not exponentially) with the dimension of the phase space.

Finally, the number of terms in the separate representation of the solution, $n_{\text{DOF}}$, can be chosen as a function of the desired level of accuracy. A vast literature exists about error estimation in this context, see for instance, [20], [28], [29], [30], [31], [32].

## Control strategy

Once the response of the robot has been adequately characterized by means of the precise form of Eq (2), a robotic hand or gripper formed by three of these “fingers” is envisaged. Different control strategies can be set up. Here, our aim is to be able to handle delicate, fragile objects without breaking them nor letting them fall.

### Control prior to contact

Of course, the first part of the control strategy, in the absence of contact, is to position the end effectors of each of the three fingers at a desired location. This is a simple example of an inverse problem mentioned before, that can be expressed as the minimization of a functional

$$\mu =\left[F_{1},F_{2}\right]=\text{arg}\;\underset{\mu^{*}=\left[F_{1}^{*},F_{2}^{*}\right]}{\text{min}}J\left(F_{1},F_{2}\right),$$

with $J\left(F_{1},F_{2}\right)=∥u\left(x_{0},\mu \right)-u_{0}∥,$ and ***u***_0_ the desired position at the end effector, located at ***x***_0_ in the reference configuration. In this case we choose μ* ∈ [0, 100]^2^ N.

### Control after contact

We assume that some tactile device has been embedded in the finger such as, for instance, a TakkTile one [33]. This type of devices provide with the contact pressure once it has occurred. The pressure at the tactile device should have been expressed in separated form as well:

$$p\left(F_{1},F_{2},d\right)\approx \sum_{i=1}^{m_{\text{mod}}}P_{i}^{1}\left(F_{1}\right)\cdot P_{i}^{2}\left(F_{2}\right)\cdot H_{i}\left(d\right),$$ (3)

with *d* the distance from the finger at rest to the contact plane, tangent to the solid at the contact point. Since the robot has actually no information on the relative position of the object to handle, this value is obtained as the position of the finger, recorded once the tactile device has informed about a non-null contact pressure. With Eq (3) the pressure contact is therefore fully characterized in a reduced-order fashion. The object to handle is assumed to have an admissible stress value *σ*, not to be reached.

The control strategy will be composed, therefore, by the following steps:

1. We approach the fingers in normal direction —by applying, say, force *F*_1_— until the tactile sensor detects contact. At this moment we determine *d* = d* by employing Eq (3).
2. From now on, assuming a certain pressure to hold the object without breaking, $p^{\text{obj}}<\sigma$, the forces in the finger will be those that minimize the functional
  $$\mu =\left[F_{1},F_{2}\right]=\text{arg}\;\underset{\mu^{*}}{\text{min}}\;L\left(\mu \right),$$ (4)
  with $L\left(\mu \right)=||p\left(F_{1},F_{2},d^{*}\right)-p^{\text{obj}}\left|\right|$.

This minimization procedure can be accomplished by the Levemberg-Marquardt algorithm, for instance, by noting that the necessary sensitivities can be computed as

$$\begin{array}{cc} \frac{\partial L}{\partial F_{1}} & =\frac{\partial }{\partial F_{1}}(p\left(F_{1},F_{2},d^{*}\right)-p^{\text{obj}}{)}^{2}\\ & =2(\sum_{i=1}^{m_{\text{mod}}}P_{i}^{1}\left(F_{1}\right)\cdot P_{i}^{2}\left(F_{2}\right)\cdot H_{i}\left(d^{*}\right)-p^{\text{obj}})\\ & \;\cdot (\sum_{i=1}^{m_{\text{mod}}}\frac{\partial P_{i}^{1}\left(F_{1}\right)}{\partial F_{1}}\cdot P_{i}^{2}\left(F_{2}\right)\cdot H_{i}\left(d^{*}\right)). \end{array}$$

Since the separated functions $P_{i}^{1}$, $P_{i}^{2}$, *H*_*i*_ are actually approximated in a finite element sense, our method stores only vectors containing the nodal values of these functions. These vectors are then multiplied or differentiated in a finite element sense with great speed and a minimum consumption of CPU time. The true advantage of our method resides actually in the separated form of the variables, displacement and pressure, at the end effector.

## Experiments and results

### Model of the finger

**Fig 2:**
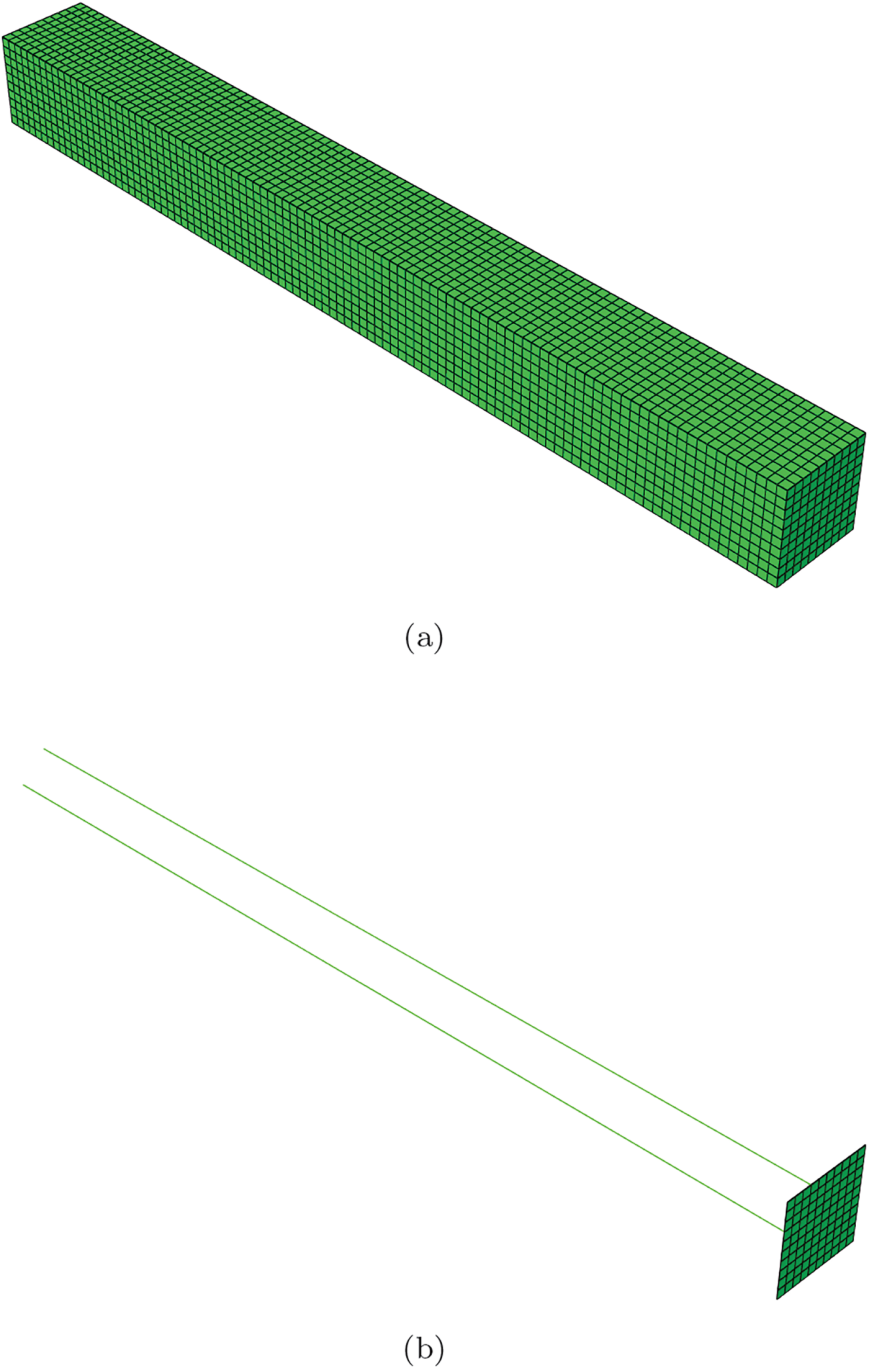
Finite element model of the finger segment. Top: discretization of the rubber envelope. Bottom: steel tendons and end plate.

Each finger is assumed to be composed by one or more modules (segments) like the one depicted in Fig 1. The rubber part of the finger is assumed to be composed by a neo-Hookean material with *C*_1_ = 1.9*MPa* and *D*_1_ = 2.43 ⋅ 10^−7^*Pa*^−1^. These values correspond to typical values of rubber-like materials. Each segment has been meshed by employing linear hexahedral finite elements. The mesh is shown in Fig 2. It is composed by 10 000 linear hexahedral elements and therefore slightly more than 30 000 degrees of freedom.

**Fig 3:**
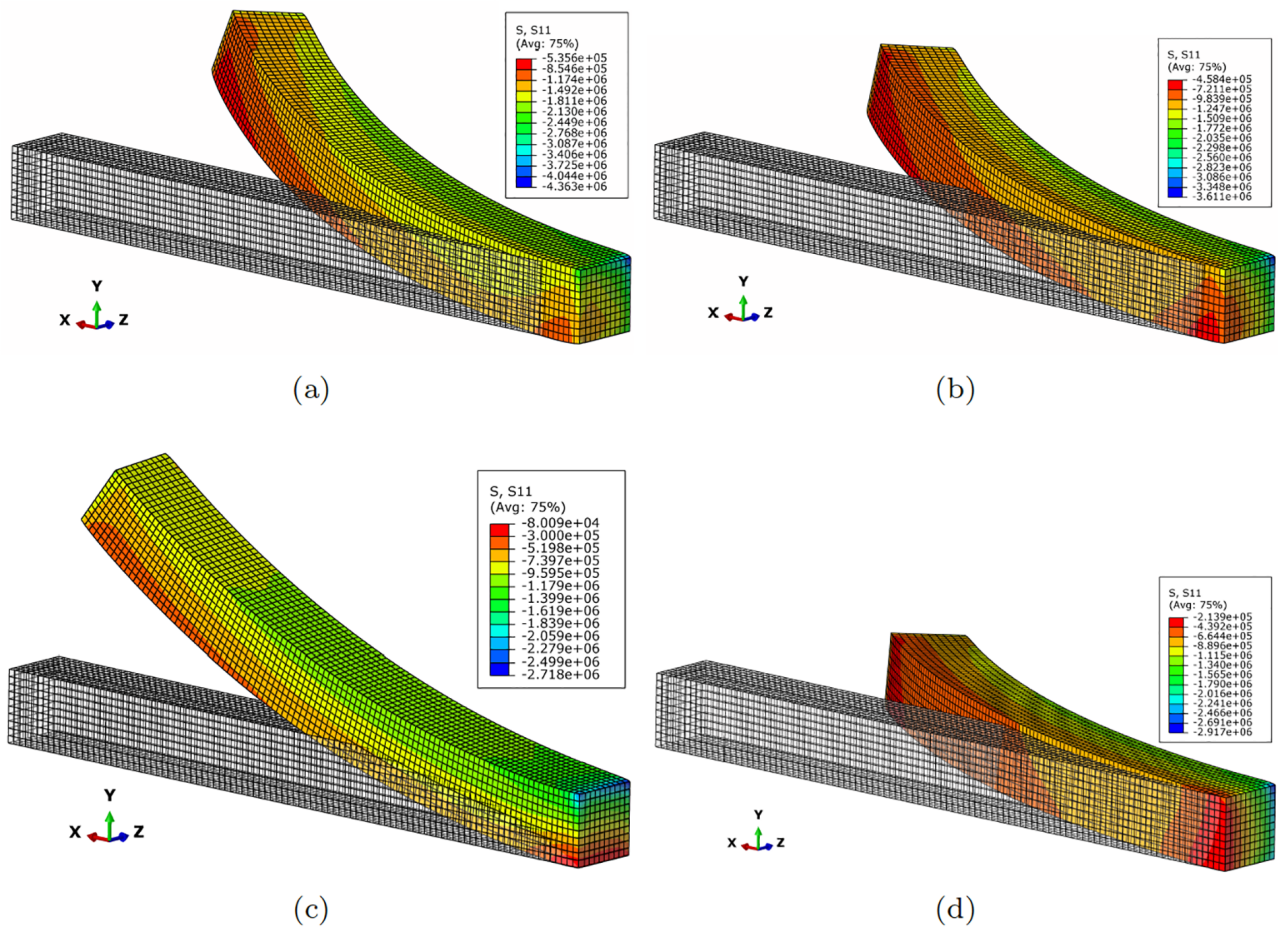
Different configurations of the finger segment. (a) *F*_1_ = 100*N*, *F*_2_ = 100*N*; (b) *F*_1_ = 50*N*, *F*_2_ = 100*N*; (c) *F*_1_ = 100*N*, *F*_2_ = 0*N*; (d) *F*_1_ = 10*N*, *F*_2_ = 100*N*. The legend corresponds to *S*_11_, the first component of the second Piola-Kirchhoff stress tensor. Symmetric configurations can be obtained by actuating the tendons situated at opposite positions.

**Fig 4:**
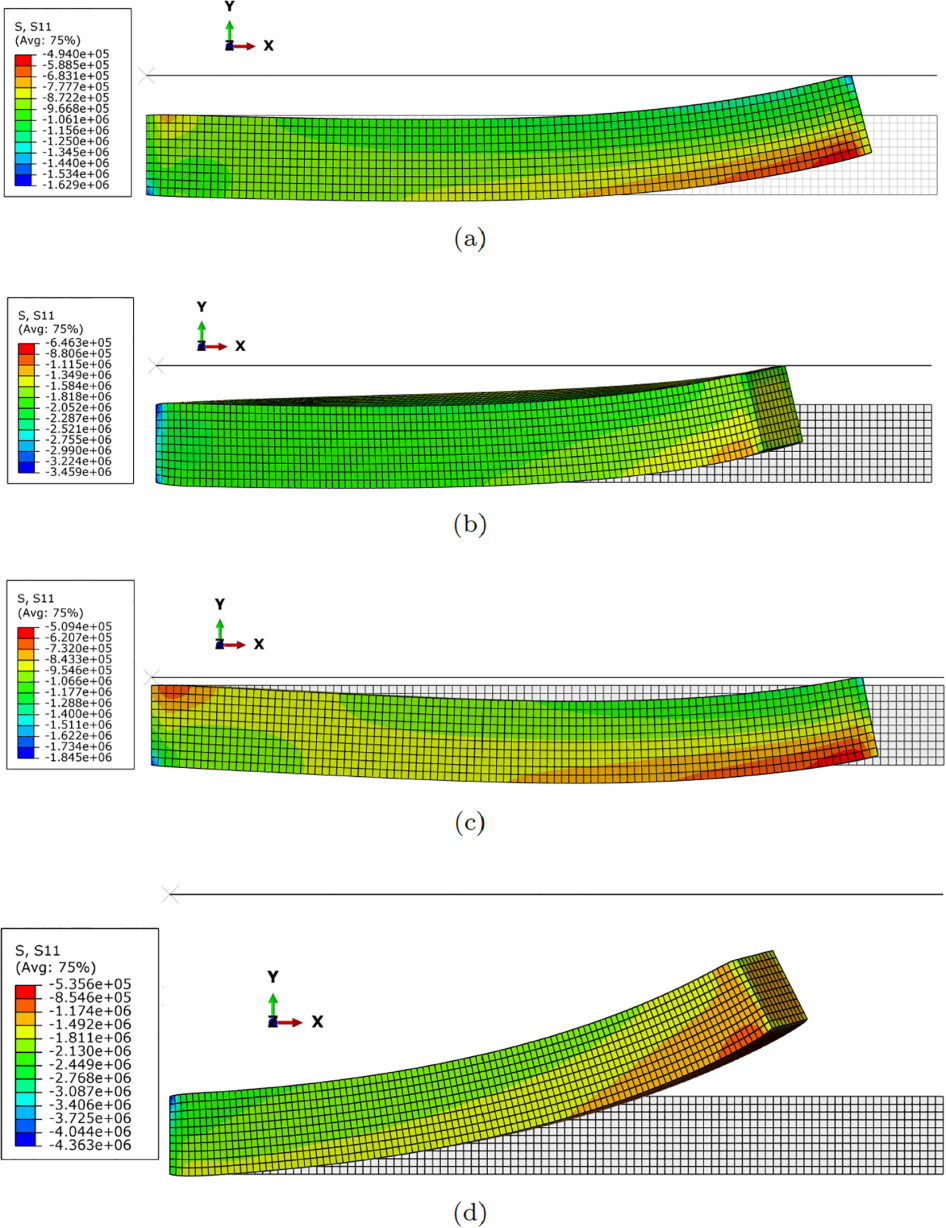
Different configurations of the finger segment after contact. (a) *F*_1_ = 100*N*, *F*_2_ = 0*N*, *d* = 5*mm*; (b) *F*_1_ = 100*N*, *F*_2_ = 100*N*, *d* = 5*mm*; (c) *F*_1_ = 100*N*, *F*_2_ = 0*N*, *d* = 1*mm*; (d) *F*_1_ = 100*N*, *F*_2_ = 100*N*, *d* = 31*mm* (no contact is observed in this particular configuration). The legend corresponds to *S*_11_, the first component of the second Piola-Kirchhoff stress tensor. Symmetric configurations can be obtained by actuating the tendons situated at opposite positions.

Fig 3 shows different configurations of the finger composed by one single segment under different actuator conditions. Similarly, Fig 4 represents different configuration once contact has occurred. It is worth noting that this figure exemplifies the way of determining the *h* distance between the robot at rest and the object, and therefore how to particularize Eq (3) to obtain the response surface of the pressure field for each particular configuration.

**Fig 5:**
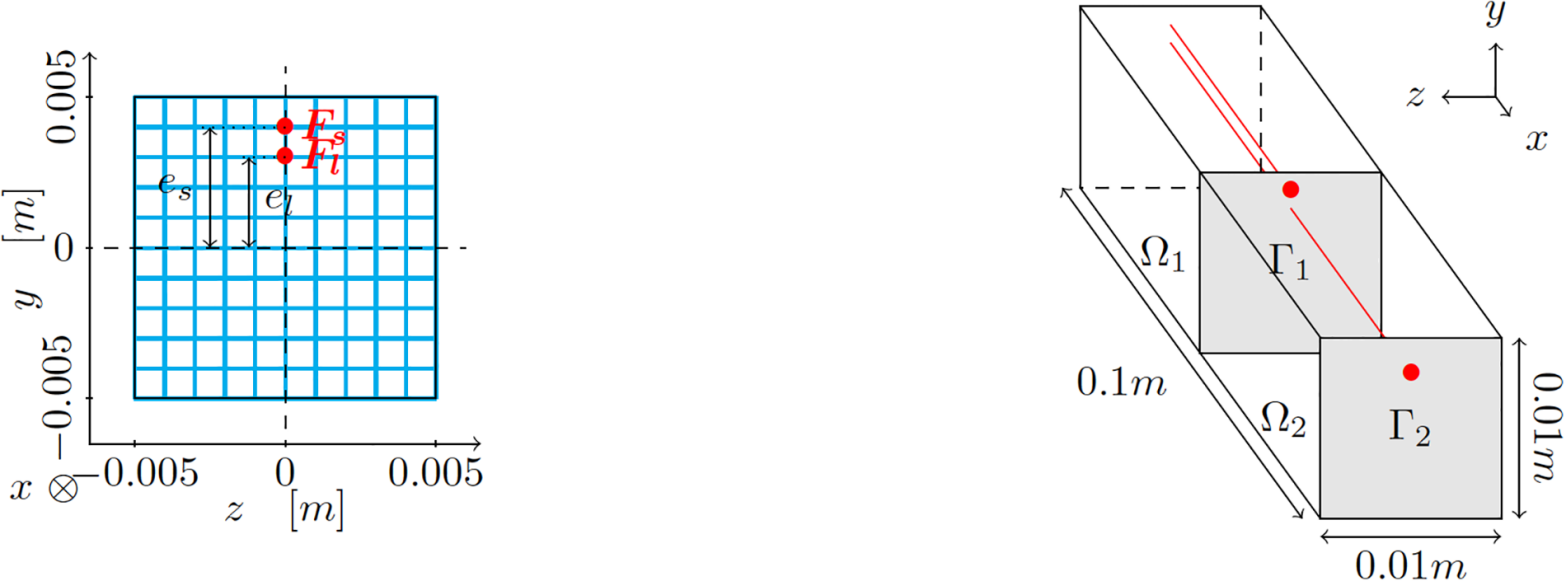
(Left) Cross sectional view of a bi-segmental finger. There are four pairs of tendons (only one is shown for clarity). The short tendons (subscript *s*) run through the finger with an eccentricity *e*_*s*_ = 0.004 m and the long ones (subscript *l*) with *e*_*l*_ = 0.003 m. (b) Three-dimensional representation of a bi-segmental finger. Both segments Ω_1_ and Ω_2_ are separated by the medial steel plate Γ_1_. The distal steel plate Γ_2_ is at the tip of the finger. The long tendon is assumed to slide freely through the medial plate Γ_1_.

**Fig 6:**
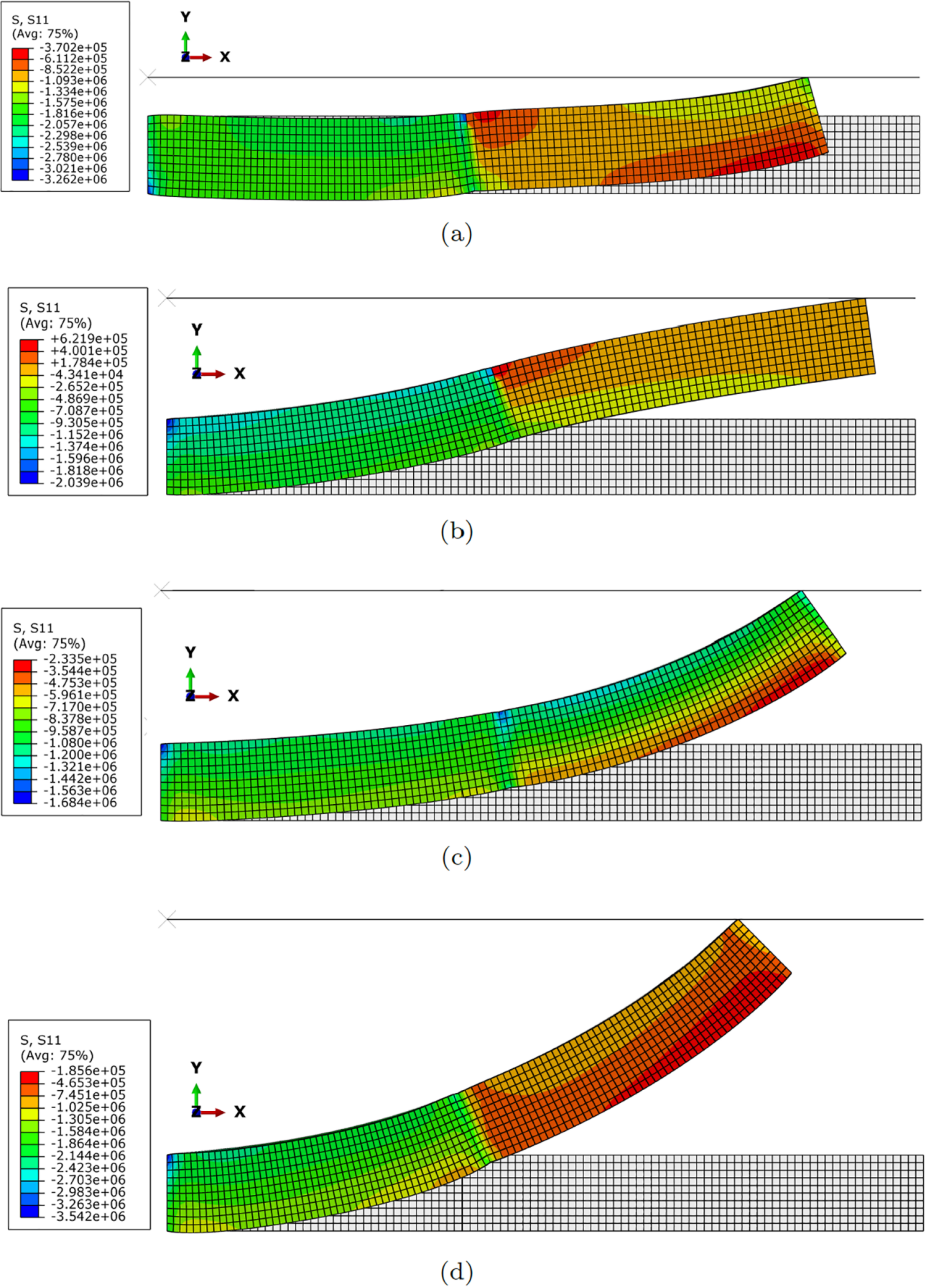
Different configurations of the finger segment after contact, when only a pair of tendons is actuated. (a) *F*_*s*_ = 100*N*, *F*_*l*_ = 100*N*, *d* = 5*mm*; (b) *F*_*s*_ = 100*N*, *F*_*l*_ = 0*N*, *d* = 16*mm*; (c) *F*_*s*_ = 0*N*, *F*_*l*_ = 100*N*, *d* = 20*mm*; (d) *F*_*s*_ = 100*N*, *F*_*l*_ = 100*N*, *d* = 31*mm*. The legend corresponds to *S*_11_, the first component of the second Piola-Kirchhoff stress tensor. Symmetric configurations can be obtained by actuating the tendons situated at opposite positions.

An extension of the model for a more sophisticated robot can be achieved by composing two segments, controlled by eight tendons, see Fig 5. In Fig 6 different configurations of this robot are shown, for different robot-to-object distance, *d*. As an obvious consequence, the number of degrees of freedom in the control algorithm increase. Results below will show, however, that the proposed methodology is able to cope with them under real-time constraints.

### Results

#### One-segment robot

We first performed a battery of tests on the robot constructed with one segment (and therefore four tendons). Starting at 10 000 randomly spaced initial positions, we computed the time to obtain the necessary forces at the two active tendons to reach the contact surface of the object and apply the necessary pressure, i.e., to solve the problem defined by Eq (4). These results were obtained by employing Matlab 2017a, running on a Mac Pro computer with four Intel Xeon E5 processors running at 3.5 GHz and are reported in Table 1.

**Table 1:** Results of the experimental campaign for the one-segment robot.

Sample size	Average time	Std dev	Min	Max
10000	7.1605 ms	1.2538 ms	5.4805 ms	31.0749 ms

**Fig 7:**
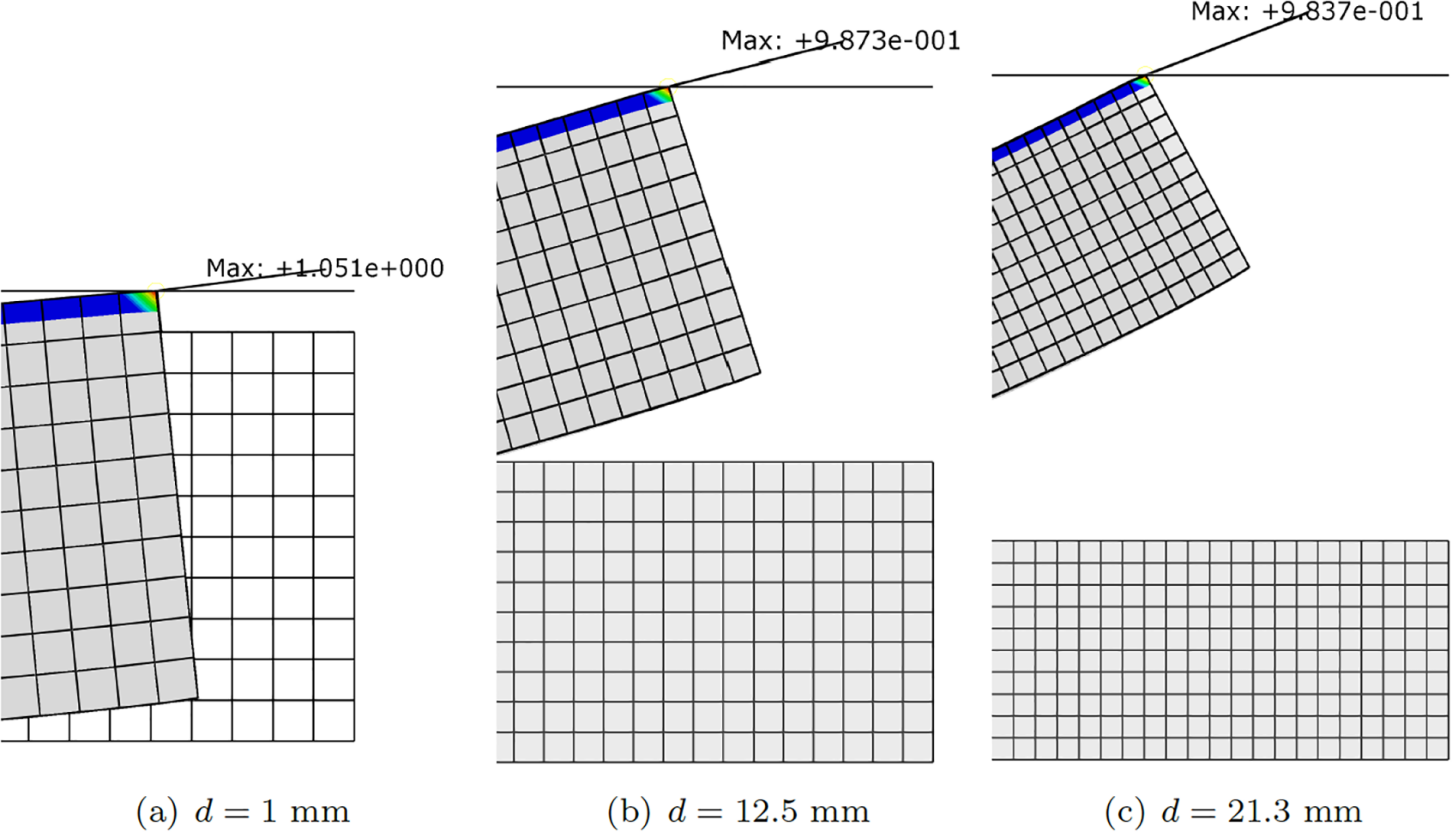
Verification of the control strategy for the one-segment robot. We took $p^{\text{obj}}=1$ N and obtained the necessary forces in the tendons by solving Eq (4). With these force values, we then ran a direct numerical simulation whose results are shown. Errors in pressure for each of the three shown cases are 5% for (a) and 2% for (b) and (c).

To verify the accuracy of the reduced-order strategy, we compared the results obtained by the control strategy with those obtained with a direct numerical simulation in which we introduce the forces in the tendons provided by the control algorithm. Errors in *L*2-norm for the displacement field and in *L*∞-norm for pressure were obtained. For the displacement field, the error found was on the order of *e*_*L*2_ ≈ 0.01%. For the pressure, this error raised to something between 3 and 5%, see Fig 7.

#### Two-segment robot

**Fig 8:**
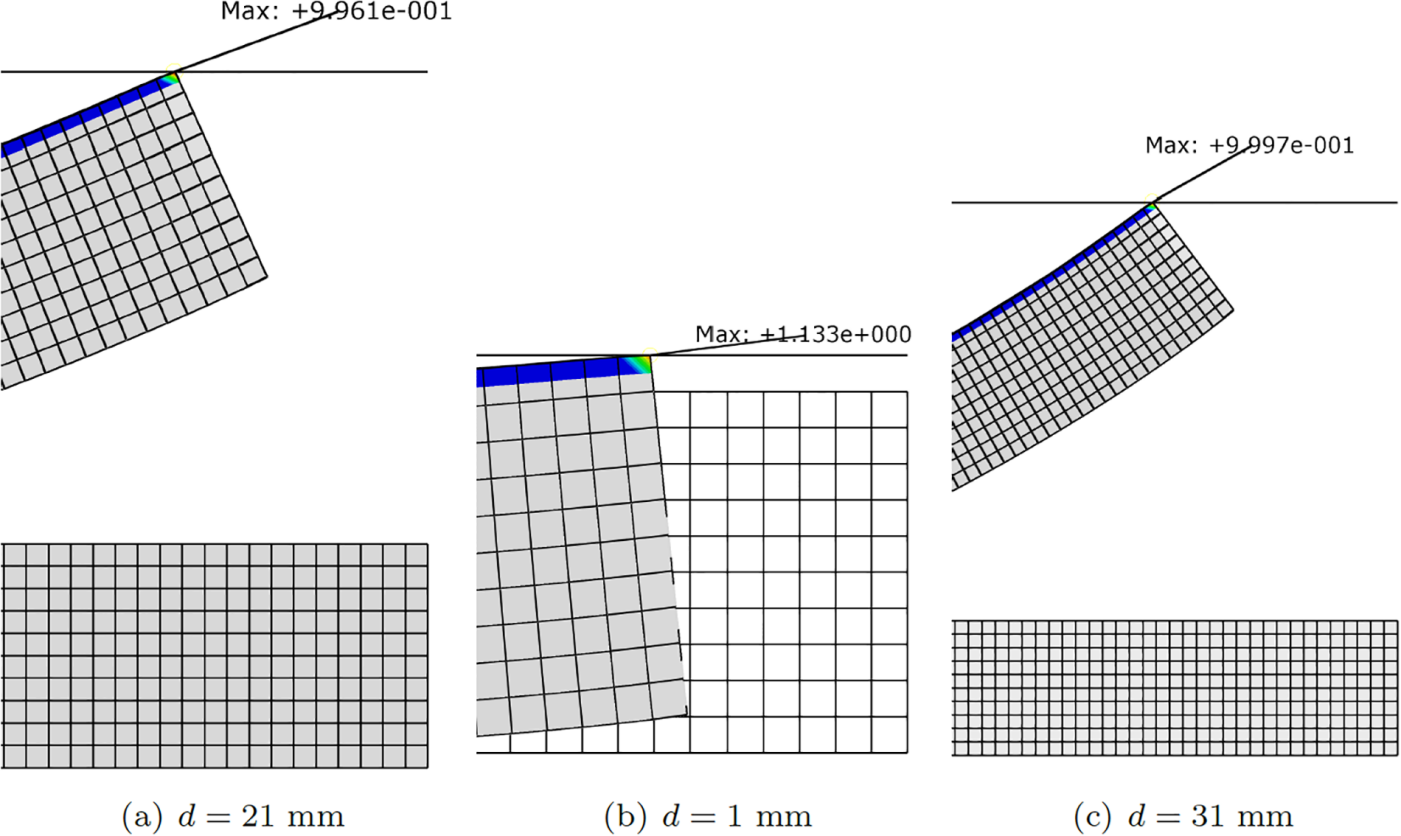
Verification of the control strategy for the two-segment robot. We took $p^{\text{obj}}=1$ N and obtained the necessary forces in the tendons by solving Eq (4). With these force values, we then ran a direct numerical simulation whose results are shown. Errors in pressure for each of the three shown cases are 4% for (a) and 13% for (b) and 0.3% for (c).

A similar experimental campaign was accomplished for the two-segment robot. In this case, the only difference with the previous section is the size of the parametric space. In this case, tests were performed again on a Mac Pro 6,1 computer equipped with Quad-core Intel Xeon E5 at 3.5, running Matlab R2017a on a single thread. In this framework, the proposed strategy was able to provide results at mean values of some 30 ms. Errors in the predicted pressure values, however, were slightly higher for one case, which can be solved nevertheless by augmenting the number of terms in Eq (2). See Fig 8 for more details on the results. The 13% error reported for one particular configuration suggests maybe the need for subsequent refinements in the meshing of the parametric space, particularly around this region. The rest of the tested values showed very limited errors, below 5%.

### Discussion

**Fig 9:**
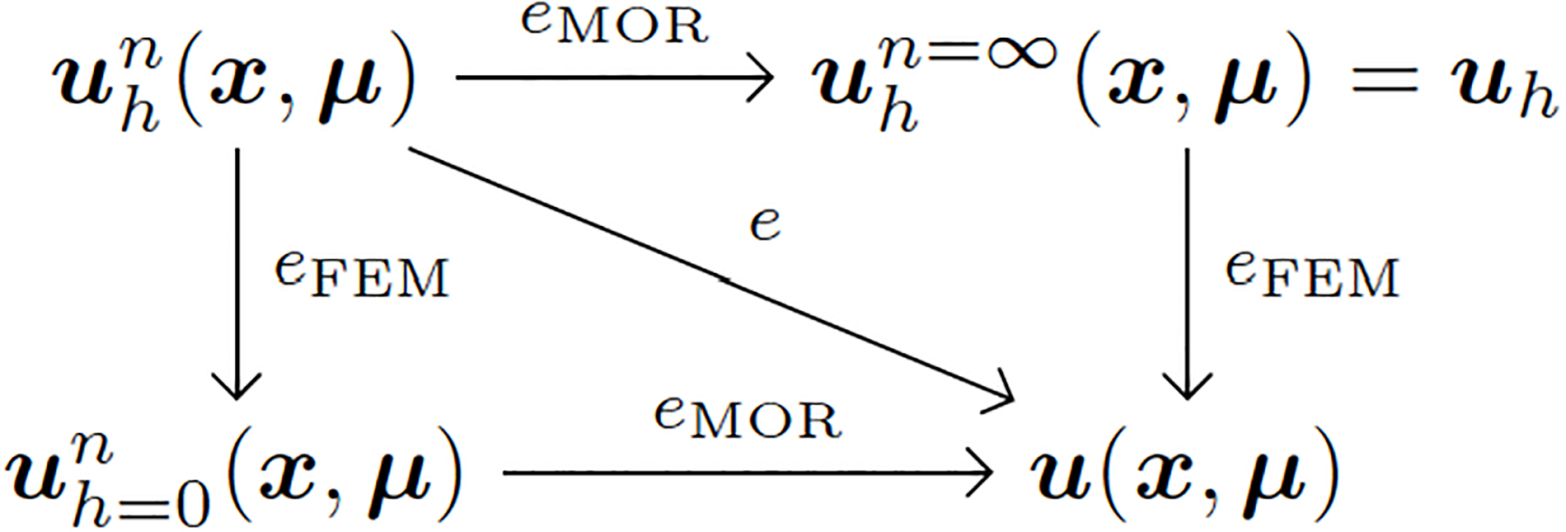
Error in a reduced order model obtained by PGD and its relationship with the approximation error due to standard finite element modeling.

The just presented results have been obtained with a model that employed 10000 elements to discretize the rubber matrix of each segment of the robot. The parametric space was discretized by employing only four elements along each dimension, which is obviously a rough discretization that can be much improved. To further improve a reduce model, two alternative routes exist. These are summarized in Fig 9, where *h* refers here to finite element size and *n* to the number of terms in the MOR approximation, see Eq (2). It can be noticed how the sources of error in the solution of the model are two-fold. On one side, the size of the finite element mesh. Of course, the finer the mesh, the better the results, and hence the error coined as *e*_FEM_. On the other, the truncation of the sum in Eq (2), adds a new source of error, here coined as *e*_MOR_. Both sources of error could ideally be separated if we take a reference solution with either a zero-sized mesh, or an infinite number of terms in the MOR solution. Both contribute to the total error of the reduced model, *e*.

Therefore, in order to improve the results, if needed, or, equivalently, minimize the error, two strategies arise: to reduce the mesh or to add more terms to the MOR approximation. In the literature, several methods exist to estimate the error and give a precise indication on how to proceed, see, for instance, [28], [32], [20]. But it is important to notice that, for a sufficiently high number of terms in the MOR approach, the model reproduces the finite element solution. Therefore, sometimes it is simply nonsense to continue augmenting the number of terms in the MOR approximation, Eq (2), since the error could be governed by a rough finite element mesh.

## References

[1] Trivedi D, Rahn CD, Kier WM, Walker ID. Soft robotics: Biological inspiration, state of the art, and future research. Applied Bionics and Biomechanics. 2008;5(3):99–117. doi: 10.1155/2008/520417

[2] Rus D, Tolley MT. Design, fabrication and control of soft robots. Nature. 2015;521(7553):467–475. doi: 10.1038/nature14543 26017446 10.1038/nature14543

[3] Pfeifer R, Lungarella M, Iida F. The Challenges Ahead for Bio-inspired ‘Soft’ Robotics. Commun ACM. 2012;55(11):76–87. doi: 10.1145/2366316.2366335

[4] Shepherd RF, Ilievski F, Choi W, Morin SA, Stokes AA, Mazzeo AD, et al Multigait soft robot. Proceedings of the National Academy of Sciences. 2011;108(51):20400–20403. doi: 10.1073/pnas.1116564108 10.1073/pnas.1116564108 PMC3251082 22123978

[5] Katzschmann RK, d Maille A, Dorhout DL, Rus D. Cyclic hydraulic actuation for soft robotic devices. In: 2016 IEEE/RSJ International Conference on Intelligent Robots and Systems (IROS); 2016. p. 3048–3055.

[6] Wriggers P. Computational contact mechanics. Springer Science & Business Media; 2006.

[7] Coevoet E, Escande A, Duriez C. Optimization-Based Inverse Model of Soft Robots With Contact Handling. IEEE Robotics and Automation Letters. 2017;2(3):1413–1419. doi: 10.1109/LRA.2017.2669367

[8] Chinesta F, Leygue A, Bordeu F, Aguado JV, Cueto E, Gonzalez D, et al PGD-Based Computational Vademecum for Efficient Design, Optimization and Control. Archives of Computational Methods in Engineering. 2013;20(1):31–59. doi: 10.1007/s11831-013-9080-x

[9] Runge G, Wiese M, Günther L, Raatz A. A framework for the kinematic modeling of soft material robots combining finite element analysis and piecewise constant curvature kinematics. In: 2017 3rd International Conference on Control, Automation and Robotics (ICCAR); 2017. p. 7–14.

[10] Gilbertson MD, McDonald G, Korinek G, de Ven JDV, Kowalewski TM. Serially Actuated Locomotion for Soft Robots in Tube-Like Environments. IEEE Robotics and Automation Letters. 2017;2(2):1140–1147. doi: 10.1109/LRA.2017.2662060

[11] Renda F, Giorelli M, Calisti M, Cianchetti M, Laschi C. Dynamic Model of a Multibending Soft Robot Arm Driven by Cables. IEEE Transactions on Robotics. 2014;30(5):1109–1122. doi: 10.1109/TRO.2014.2325992

[12] Bonet J, Wood RD. Nonlinear continuum mechanics for finite element analysis. Cambridge University Press; 2008.

[13] Laschi C, Cianchetti M, Mazzolai B, Margheri L, Follador M, Dario P. Soft Robot Arm Inspired by the Octopus. Advanced Robotics. 2012;26(7):709–727. doi: 10.1163/156855312X626343 10.1088/1748-3182/7/2/025005 22617166

[14] Roßmann J, Schluse M, Rast M, Kaigom EG, Cichon T, Schluse M. In: Verl A, Albu-Schäffer A, Brock O, Raatz A, editors. Simulation Technology for Soft Robotics Applications. Berlin, Heidelberg: Springer Berlin Heidelberg; 2015 p. 100–119. Available from: http://dx.doi.org/10.1007/978-3-662-44506-8_10.

[15] Duriez C, Duriez C. Finite Element Method Control of Elastic Soft Robots based on Real-Time Finite Element Method. In: 2013 IEEE International Conference on Robotics and Automation (ICRA). IEEE Computer Society; 2013. p. 3967–3972.

[16] Largilliere F, Verona V, Coevoet E, Sanz-Lopez M, Dequidt J, Duriez C. Real-time control of soft-robots using asynchronous finite element modeling. 2015 IEEE International Conference on Robotics and Automation (ICRA). 2015; p. 2550–2555.

[17] Gravagne I, Rahn CD, Walker ID. Large Deflection Dynamics and Control for Planar Continuum Robots. IEEE/ASME Transactions on Mechatronics. 2003;8(2):299–307. doi: 10.1109/TMECH.2003.812829

[18] Chinesta F, Ladevèze P. Separated Representations and PGD-Based Model Reduction. Springer; 2014.

[19] Hesthaven J, Rozza G, Stamm B. Certified reduced basis methods for parametrized partial differential equations. Springer Verlag; 2015.

[20] Rozza G, Huynh DBP, Patera AT. Reduced basis approximation and a posteriori error estimation for affinely parametrized elliptic coercive partial differential equations—application to transport and continuum mechanics. Archives of Computational Methods in Engineering. 2008;15/3:229–275. doi: 10.1007/s11831-008-9019-9

[21] Chinesta F, Ammar A, Cueto E. Recent advances in the use of the Proper Generalized Decomposition for solving multidimensional models. Archives of Computational Methods in Engineering. 2010;17(4):327–350. doi: 10.1007/s11831-010-9049-y

[22] Ryckelynck D, Chinesta F, Cueto E, Ammar A. On the a priori Model Reduction: Overview and recent developments. Archives of Computational Methods in Engineering. 2006;12(1):91–128. doi: 10.1007/BF02905932

[23] Karhunen K. Uber lineare methoden in der wahrscheinlichkeitsrechnung. Ann Acad Sci Fennicae, ser Al Math Phys. 1946;37.

[24] Loève MM. Probability theory The University Series in Higher Mathematics, 3rd ed Van Nostrand, Princeton, NJ; 1963.

[25] Quarteroni A, Rozza G, Manzoni A. Certified reduced basis approximation for parametrized PDE and applications. J Math in Industry. 2011;3.

[26] Chinesta F, Cueto E. PGD-Based Modeling of Materials, Structures and Processes. Springer International Publishing Switzerland; 2014.

[27] Cueto E, González D, Alfaro I. Proper Generalized Decompositions: An Introduction to Computer Implementation with Matlab SpringerBriefs in Applied Sciences and Technology. Springer International Publishing; 2016 Available from: https://books.google.es/books?id=M9eHDAAAQBAJ.

[28] Ammar A, Chinesta F, Diez P, Huerta A. An error estimator for separated representations of highly multidimensional models. Computer Methods in Applied Mechanics and Engineering. 2010;199(25-28):1872–1880. doi: 10.1016/j.cma.2010.02.012

[29] Ladeveze P, Chamoin L. On the verification of model reduction methods based on the proper generalized decomposition. Computer Methods in Applied Mechanics and Engineering. 2011;200(23-24):2032–2047. doi: 10.1016/j.cma.2011.02.019

[30] Moitinho de Almeida JP. A basis for bounding the errors of proper generalised decomposition solutions in solid mechanics. International Journal for Numerical Methods in Engineering. 2013;94(10):961–984. doi: 10.1002/nme.4490

[31] Patera AT, Rozza G. Reduced Basis Approximation and A Posteriori Error Estimation for Parametrized Partial Differential Equations MIT Pappalardo Monographs in Mechanical Engineering; 2007.

[32] Alfaro I, Gonzalez D, Zlotnik S, Diez P, Cueto E, Chinesta F. An error estimator for real-time simulators based on model order reduction. Advanced Modeling and Simulation in Engineering Sciences. 2015;2(1):30 doi: 10.1186/s40323-015-0050-8

[33] Jentoft LP, Tenzer Y, Vogt D, Liu J, Wood RJ, Howe RD. Flexible, stretchable tactile arrays from MEMS barometers. In: 2013 16th International Conference on Advanced Robotics (ICAR); 2013. p. 1–6.

